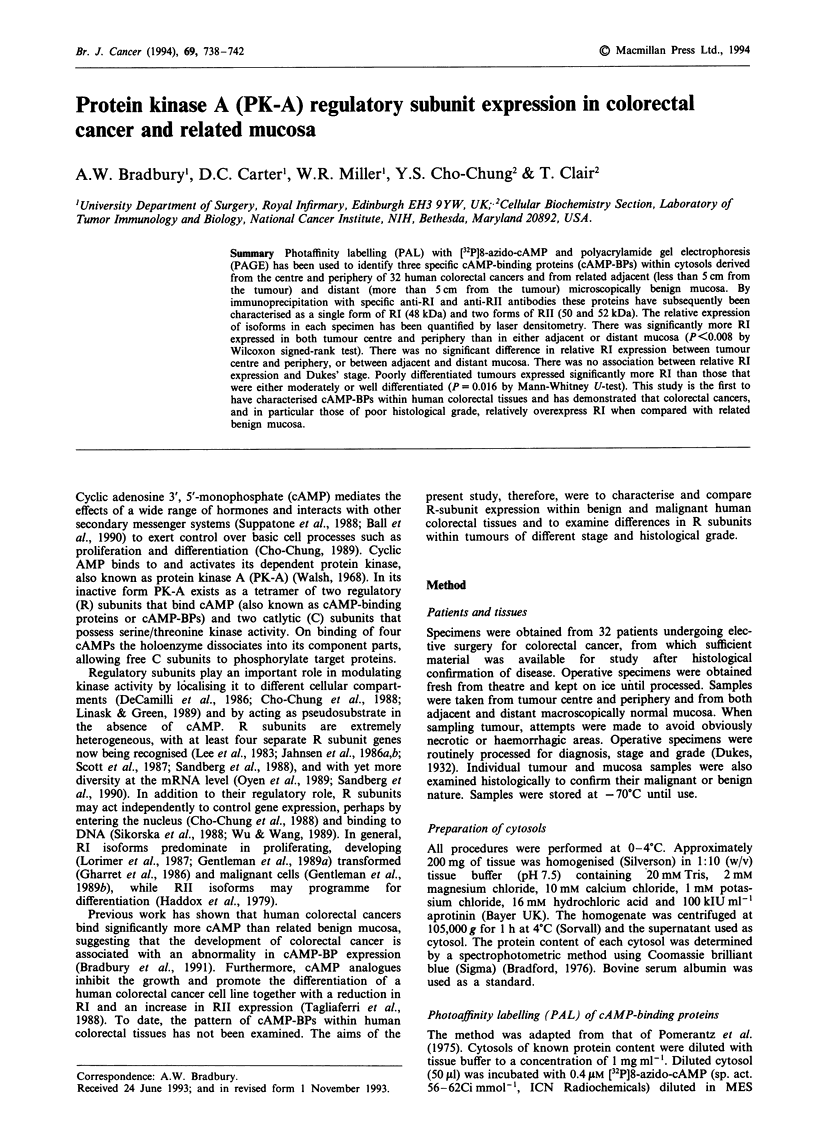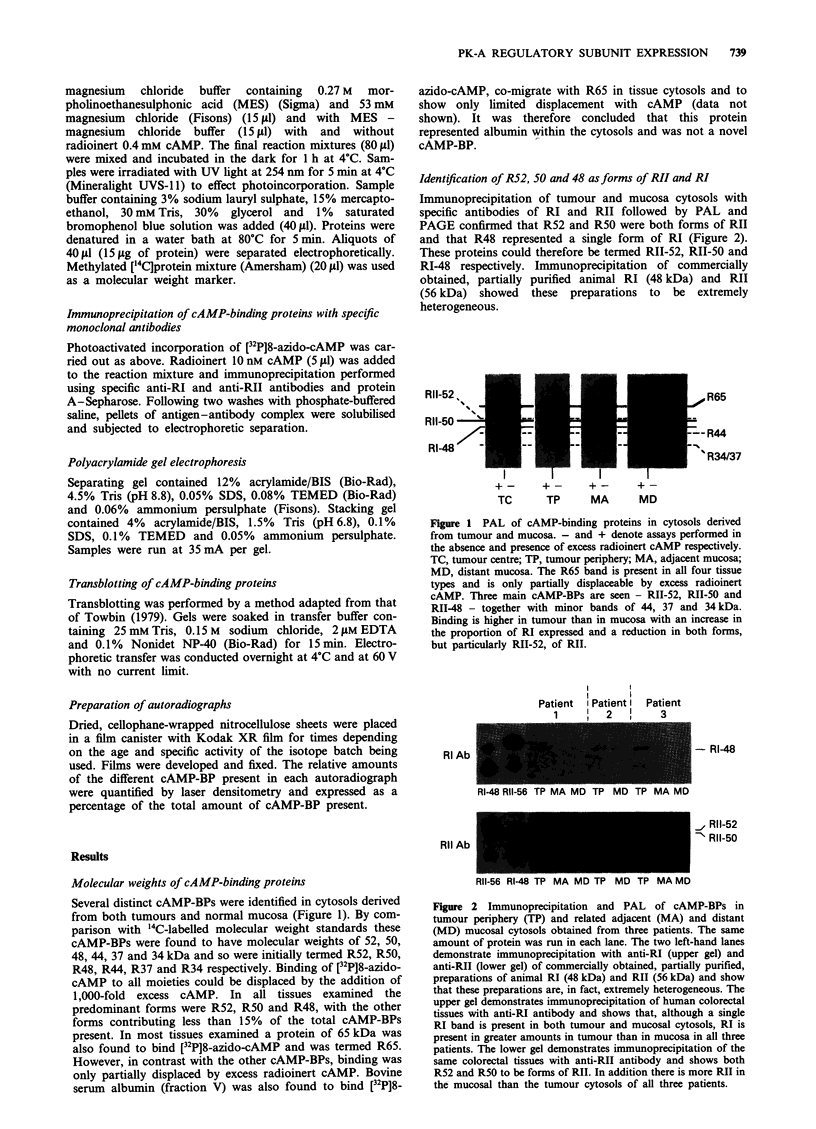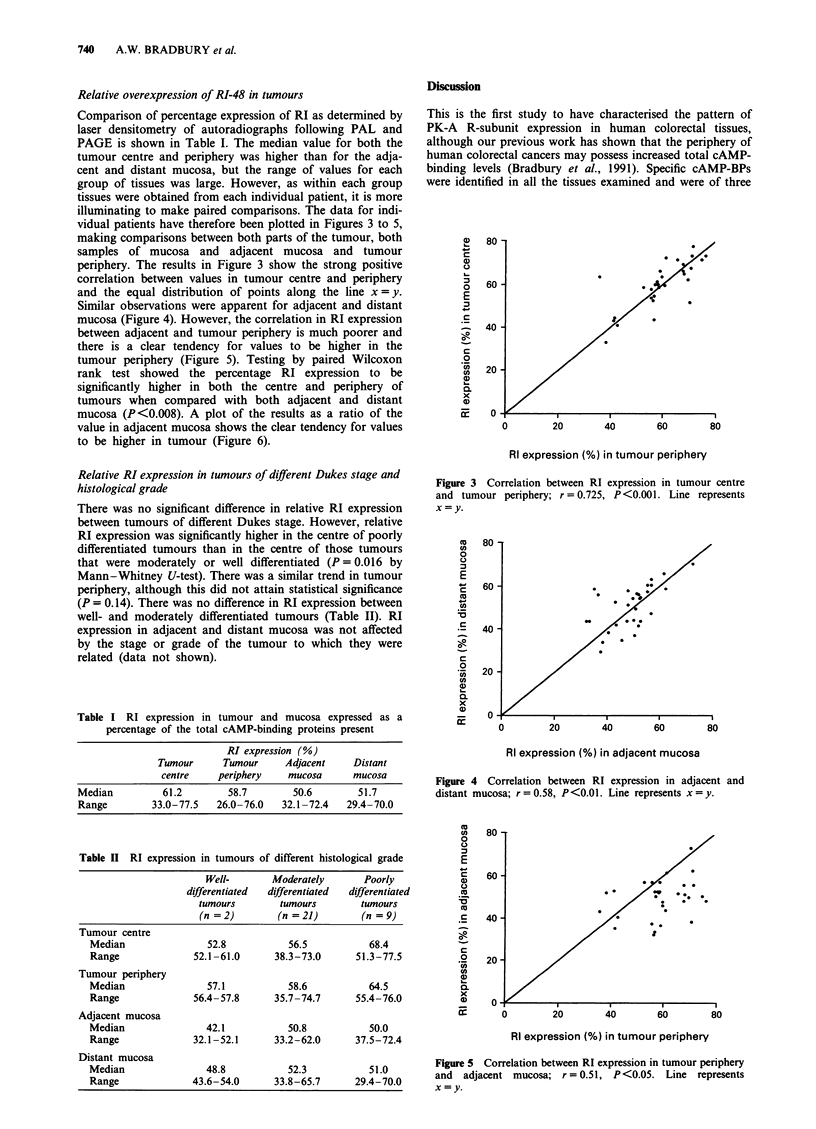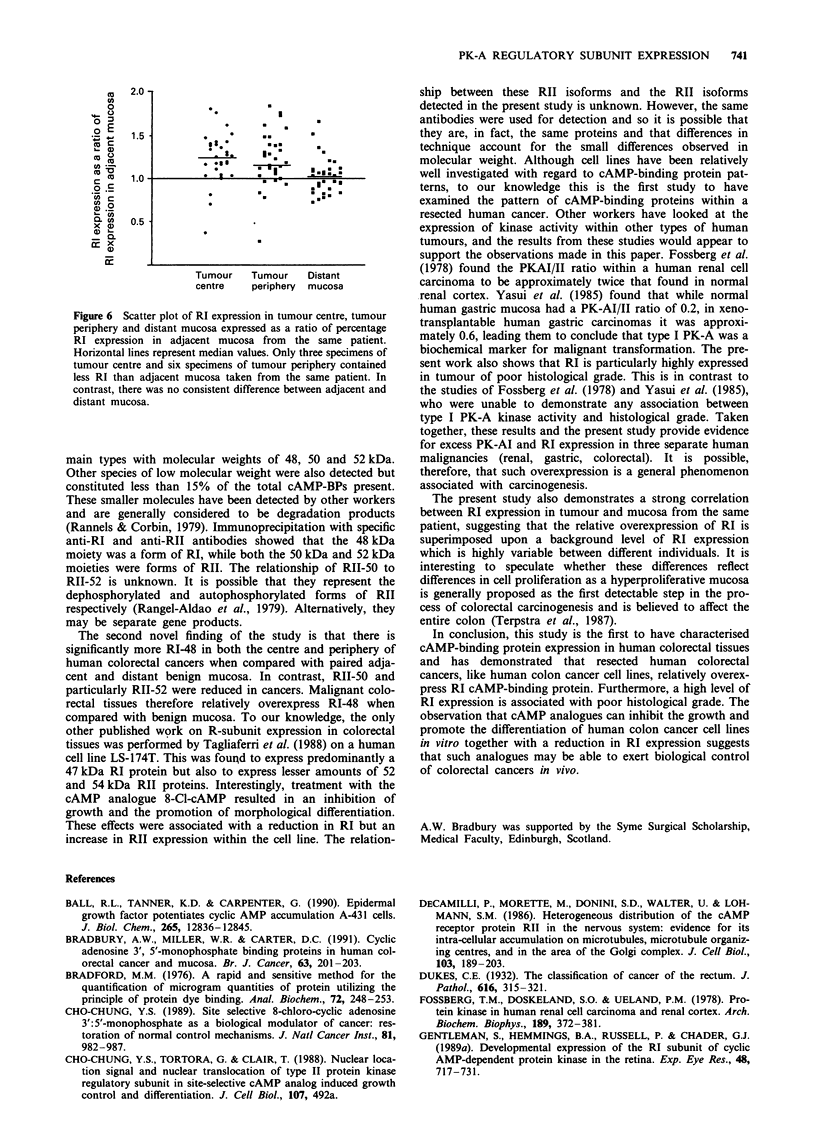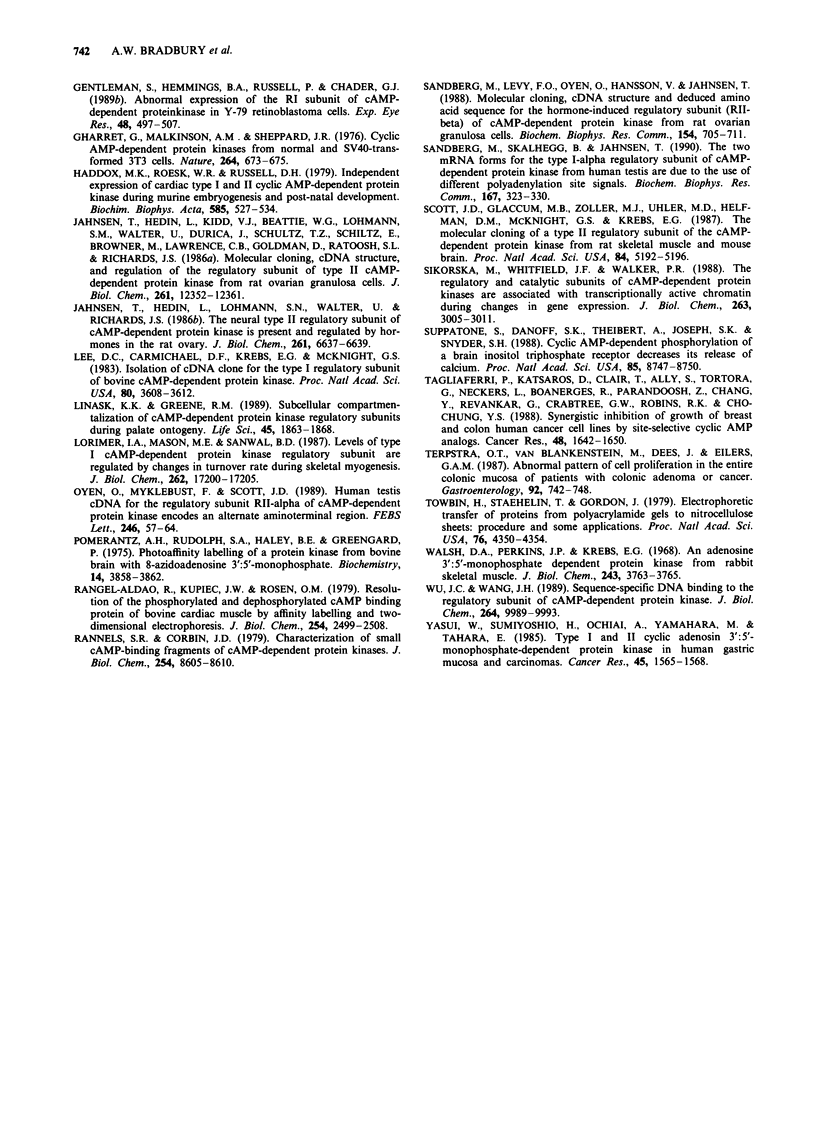# Protein kinase A (PK-A) regulatory subunit expression in colorectal cancer and related mucosa.

**DOI:** 10.1038/bjc.1994.139

**Published:** 1994-04

**Authors:** A. W. Bradbury, D. C. Carter, W. R. Miller, Y. S. Cho-Chung, T. Clair

**Affiliations:** University Department of Surgery, Royal Infirmary, Edinburgh, UK.

## Abstract

**Images:**


Br. J. Cancer (1994), 69, 738-742 © Macmillan Press Ltd., 1994

### Protein kinase A (PK-A) regulatory subunit expression in colorectal cancer and related mucosa

A.W. Bradbury[1], D.C. Carter[1], W.R. Miller[1], Y.S. Cho-Chung[2] & T. Clair[2]

[1]*University Department of Surgery, Royal Infirmary, Edinburgh EH3 9YW, UK;* [2]*Cellular Biochemistry Section, Laboratory of Tumor Immunology and Biology, National Cancer Institute, NIH, Bethesda, Maryland 20892, USA.*

**Summary** Photaffinity labelling (PAL) with [$^{32}$P]8-azido-cAMP and polyacrylamide gel electrophoresis (PAGE) has been used to identify three specific cAMP-binding proteins (cAMP-BPs) within cytosols derived from the centre and periphery of 32 human colorectal cancers and from related adjacent (less than 5 cm from the tumour) and distant (more than 5 cm from the tumour) microscopically benign mucosa. By immunoprecipitation with specific anti-RI and anti-RII antibodies these proteins have subsequently been characterised as a single form of RI (48 kDa) and two forms of RII (50 and 52 kDa). The relative expression of isoforms in each specimen has been quantified by laser densitometry. There was significantly more RI expressed in both tumour centre and periphery than in either adjacent or distant mucosa ($P<0.008$ by Wilcoxon signed-rank test). There was no significant difference in relative RI expression between tumour centre and periphery, or between adjacent and distant mucosa. There was no association between relative RI expression and Dukes' stage. Poorly differentiated tumours expressed significantly more RI than those that were either moderately or well differentiated ($P = 0.016$ by Mann-Whitney $U$-test). This study is the first to have characterised cAMP-BPs within human colorectal tissues and has demonstrated that colorectal cancers, and in particular those of poor histological grade, relatively overexpress RI when compared with related benign mucosa.

Cyclic adenosine 3′, 5′-monophosphate (cAMP) mediates the effects of a wide range of hormones and interacts with other secondary messenger systems (Suppatone *et al.*, 1988; Ball *et al.*, 1990) to exert control over basic cell processes such as proliferation and differentiation (Cho-Chung, 1989). Cyclic AMP binds to and activates its dependent protein kinase, also known as protein kinase A (PK-A) (Walsh, 1968). In its inactive form PK-A exists as a tetramer of two regulatory (R) subunits that bind cAMP (also known as cAMP-binding proteins or cAMP-BPs) and two catlytic (C) subunits that possess serine/threonine kinase activity. On binding of four cAMPs the holoenzyme dissociates into its component parts, allowing free C subunits to phosphorylate target proteins.

Regulatory subunits play an important role in modulating kinase activity by localising it to different cellular compartments (DeCamilli *et al.*, 1986; Cho-Chung *et al.*, 1988; Linask & Green, 1989) and by acting as pseudosubstrate in the absence of cAMP. R subunits are extremely heterogeneous, with at least four separate R subunit genes now being recognised (Lee *et al.*, 1983; Jahnsen *et al.*, 1986*a,b*; Scott *et al.*, 1987; Sandberg *et al.*, 1988), and with yet more diversity at the mRNA level (Oyen *et al.*, 1989; Sandberg *et al.*, 1990). In addition to their regulatory role, R subunits may act independently to control gene expression, perhaps by entering the nucleus (Cho-Chung *et al.*, 1988) and binding to DNA (Sikorska *et al.*, 1988; Wu & Wang, 1989). In general, RI isoforms predominate in proliferating, developing (Lorimer *et al.*, 1987; Gentleman *et al.*, 1989*a*) transformed (Gharret *et al.*, 1986) and malignant cells (Gentleman *et al.*, 1989*b*), while RII isoforms may programme for differentiation (Haddox *et al.*, 1979).

Previous work has shown that human colorectal cancers bind significantly more cAMP than related benign mucosa, suggesting that the development of colorectal cancer is associated with an abnormality in cAMP-BP expression (Bradbury *et al.*, 1991). Furthermore, cAMP analogues inhibit the growth and promote the differentiation of a human colorectal cancer cell line together with a reduction in RI and an increase in RII expression (Tagliaferri *et al.*, 1988). To date, the pattern of cAMP-BPs within human colorectal tissues has not been examined. The aims of the present study, therefore, were to characterise and compare R-subunit expression within benign and malignant human colorectal tissues and to examine differences in R subunits within tumours of different stage and histological grade.

Correspondence: A.W. Bradbury.
Received 24 June 1993; and in revised form 1 November 1993.

### Method

### *Patients and tissues*

Specimens were obtained from 32 patients undergoing elective surgery for colorectal cancer, from which sufficient material was available for study after histological confirmation of disease. Operative specimens were obtained fresh from theatre and kept on ice until processed. Samples were taken from tumour centre and periphery and from both adjacent and distant macroscopically normal mucosa. When sampling tumour, attempts were made to avoid obviously necrotic or haemorrhagic areas. Operative specimens were routinely processed for diagnosis, stage and grade (Dukes, 1932). Individual tumour and mucosa samples were also examined histologically to confirm their malignant or benign nature. Samples were stored at −70°C until use.

### *Preparation of cytosols*

All procedures were performed at 0–4°C. Approximately 200 mg of tissue was homogenised (Silverson) in 1:10 (w/v) tissue buffer (pH 7.5) containing 20 mM Tris, 2 mM magnesium chloride, 10 mM calcium chloride, 1 mM potassium chloride, 16 mM hydrochloric acid and 100 kIU ml$^{-1}$ aprotinin (Bayer UK). The homogenate was centrifuged at 105,000 *g* for 1 h at 4°C (Sorvall) and the supernatant used as cytosol. The protein content of each cytosol was determined by a spectrophotometric method using Coomassie brilliant blue (Sigma) (Bradford, 1976). Bovine serum albumin was used as a standard.

### *Photoaffinity labelling (PAL) of cAMP-binding proteins*

The method was adapted from that of Pomerantz *et al.* (1975). Cytosols of known protein content were diluted with tissue buffer to a concentration of 1 mg ml$^{-1}$. Diluted cytosol (50 µl) was incubated with 0.4 µM [$^{32}$P]8-azido-cAMP (sp. act. 56–62Ci mmol$^{-1}$, ICN Radiochemicals) diluted in MES

magnesium chloride buffer containing 0.27 M morpholinoethanesulphonic acid (MES) (Sigma) and 53 mM magnesium chloride (Fisons) (15 µl) and with MES – magnesium chloride buffer (15 µl) with and without radioinert 0.4 mM cAMP. The final reaction mixtures (80 µl) were mixed and incubated in the dark for 1 h at 4°C. Samples were irradiated with UV light at 254 nm for 5 min at 4°C (Mineralight UVS-11) to effect photoincorporation. Sample buffer containing 3% sodium lauryl sulphate, 15% mercaptoethanol, 30 mM Tris, 30% glycerol and 1% saturated bromophenol blue solution was added (40 µl). Proteins were denatured in a water bath at 80°C for 5 min. Aliquots of 40 µl (15 µg of protein) were separated electrophoretically. Methylated [$^{14}$C]protein mixture (Amersham) (20 µl) was used as a molecular weight marker.

*Immunoprecipitation of cAMP-binding proteins with specific monoclonal antibodies*

Photoactivated incorporation of [$^{32}$P]8-azido-cAMP was carried out as above. Radioinert 10 nM cAMP (5 µl) was added to the reaction mixture and immunoprecipitation performed using specific anti-RI and anti-RII antibodies and protein A-Sepharose. Following two washes with phosphate-buffered saline, pellets of antigen-antibody complex were solubilised and subjected to electrophoretic separation.

*Polyacrylamide gel electrophoresis*

Separating gel contained 12% acrylamide/BIS (Bio-Rad), 4.5% Tris (pH 8.8), 0.05% SDS, 0.08% TEMED (Bio-Rad) and 0.06% ammonium persulphate (Fisons). Stacking gel contained 4% acrylamide/BIS, 1.5% Tris (pH 6.8), 0.1% SDS, 0.1% TEMED and 0.05% ammonium persulphate. Samples were run at 35 mA per gel.

*Transblotting of cAMP-binding proteins*

Transblotting was performed by a method adapted from that of Towbin (1979). Gels were soaked in transfer buffer containing 25 mM Tris, 0.15 M sodium chloride, 2 µM EDTA and 0.1% Nonidet NP-40 (Bio-Rad) for 15 min. Electrophoretic transfer was conducted overnight at 4°C and at 60 V with no current limit.

*Preparation of autoradiographs*

Dried, cellophane-wrapped nitrocellulose sheets were placed in a film canister with Kodak XR film for times depending on the age and specific activity of the isotope batch being used. Films were developed and fixed. The relative amounts of the different cAMP-BP present in each autoradiograph were quantified by laser densitometry and expressed as a percentage of the total amount of cAMP-BP present.

### Results

*Molecular weights of cAMP-binding proteins*

Several distinct cAMP-BPs were identified in cytosols derived from both tumours and normal mucosa (Figure 1). By comparison with $^{14}$C-labelled molecular weight standards these cAMP-BPs were found to have molecular weights of 52, 50, 48, 44, 37 and 34 kDa and so were initially termed R52, R50, R48, R44, R37 and R34 respectively. Binding of [$^{32}$P]8-azido-cAMP to all moieties could be displaced by the addition of 1,000-fold excess cAMP. In all tissues examined the predominant forms were R52, R50 and R48, with the other forms contributing less than 15% of the total cAMP-BPs present. In most tissues examined a protein of 65 kDa was also found to bind [$^{32}$P]8-azido-cAMP and was termed R65. However, in contrast with the other cAMP-BPs, binding was only partially displaced by excess radioinert cAMP. Bovine serum albumin (fraction V) was also found to bind [$^{32}$P]8-azido-cAMP, co-migrate with R65 in tissue cytosols and to show only limited displacement with cAMP (data not shown). It was therefore concluded that this protein represented albumin within the cytosols and was not a novel cAMP-BP.

*Identification of R52, 50 and 48 as forms of RII and RI*

Immunoprecipitation of tumour and mucosa cytosols with specific antibodies of RI and RII followed by PAL and PAGE confirmed that R52 and R50 were both forms of RII and that R48 represented a single form of RI (Figure 2). These proteins could therefore be termed RII-52, RII-50 and RI-48 respectively. Immunoprecipitation of commercially obtained, partially purified animal RI (48 kDa) and RII (56 kDa) showed these preparations to be extremely heterogeneous.

**Figure 1** PAL of cAMP-binding proteins in cytosols derived from tumour and mucosa. − and + denote assays performed in the absence and presence of excess radioinert cAMP respectively. TC, tumour centre; TP, tumour periphery; MA, adjacent mucosa; MD, distant mucosa. The R65 band is present in all four tissue types and is only partially displaceable by excess radioinert cAMP. Three main cAMP-BPs are seen – RII-52, RII-50 and RII-48 – together with minor bands of 44, 37 and 34 kDa. Binding is higher in tumour than in mucosa with an increase in the proportion of RI expressed and a reduction in both forms, but particularly RII-52, of RII.

**Figure 2** Immunoprecipitation and PAL of cAMP-BPs in tumour periphery (TP) and related adjacent (MA) and distant (MD) mucosal cytosols obtained from three patients. The same amount of protein was run in each lane. The two left-hand lanes demonstrate immunoprecipitation with anti-RI (upper gel) and anti-RII (lower gel) of commercially obtained, partially purified, preparations of animal RI (48 kDa) and RII (56 kDa) and show that these preparations are, in fact, extremely heterogeneous. The upper gel demonstrates immunoprecipitation of human colorectal tissues with anti-RI antibody and shows that, although a single RI band is present in both tumour and mucosal cytosols, RI is present in greater amounts in tumour than in mucosa in all three patients. The lower gel demonstrates immunoprecipitation of the same colorectal tissues with anti-RII antibody and shows both R52 and R50 to be forms of RII. In addition there is more RII in the mucosal than the tumour cytosols of all three patients.

### *Relative overexpression of RI-48 in tumours*

Comparison of percentage expression of RI as determined by laser densitometry of autoradiographs following PAL and PAGE is shown in Table I. The median value for both the tumour centre and periphery was higher than for the adjacent and distant mucosa, but the range of values for each group of tissues was large. However, as within each group tissues were obtained from each individual patient, it is more illuminating to make paired comparisons. The data for individual patients have therefore been plotted in Figures 3 to 5, making comparisons between both parts of the tumour, both samples of mucosa and adjacent mucosa and tumour periphery. The results in Figure 3 show the strong positive correlation between values in tumour centre and periphery and the equal distribution of points along the line $x = y$. Similar observations were apparent for adjacent and distant mucosa (Figure 4). However, the correlation in RI expression between adjacent and tumour periphery is much poorer and there is a clear tendency for values to be higher in the tumour periphery (Figure 5). Testing by paired Wilcoxon rank test showed the percentage RI expression to be significantly higher in both the centre and periphery of tumours when compared with both adjacent and distant mucosa ($P < 0.008$). A plot of the results as a ratio of the value in adjacent mucosa shows the clear tendency for values to be higher in tumour (Figure 6).

### *Relative RI expression in tumours of different Dukes stage and histological grade*

There was no significant difference in relative RI expression between tumours of different Dukes stage. However, relative RI expression was significantly higher in the centre of poorly differentiated tumours than in the centre of those tumours that were moderately or well differentiated ($P = 0.016$ by Mann-Whitney $U$-test). There was a similar trend in tumour periphery, although this did not attain statistical significance ($P = 0.14$). There was no difference in RI expression between well- and moderately differentiated tumours (Table II). RI expression in adjacent and distant mucosa was not affected by the stage or grade of the tumour to which they were related (data not shown).

**Table I** RI expression in tumour and mucosa expressed as a percentage of the total cAMP-binding proteins present

| | RI expression (%) | | | |
|---|---|---|---|---|
| | *Tumour centre* | *Tumour periphery* | *Adjacent mucosa* | *Distant mucosa* |
| Median | 61.2 | 58.7 | 50.6 | 51.7 |
| Range | 33.0-77.5 | 26.0-76.0 | 32.1-72.4 | 29.4-70.0 |

**Table II** RI expression in tumours of different histological grade

| | *Well-differentiated tumours (n = 2)* | *Moderately differentiated tumours (n = 21)* | *Poorly differentiated tumours (n = 9)* |
|---|---|---|---|
| Tumour centre | | | |
| Median | 52.8 | 56.5 | 68.4 |
| Range | 52.1-61.0 | 38.3-73.0 | 51.3-77.5 |
| Tumour periphery | | | |
| Median | 57.1 | 58.6 | 64.5 |
| Range | 56.4-57.8 | 35.7-74.7 | 55.4-76.0 |
| Adjacent mucosa | | | |
| Median | 42.1 | 50.8 | 50.0 |
| Range | 32.1-52.1 | 33.2-62.0 | 37.5-72.4 |
| Distant mucosa | | | |
| Median | 48.8 | 52.3 | 51.0 |
| Range | 43.6-54.0 | 33.8-65.7 | 29.4-70.0 |

### Discussion

This is the first study to have characterised the pattern of PK-A R-subunit expression in human colorectal tissues, although our previous work has shown that the periphery of human colorectal cancers may possess increased total cAMP-binding levels (Bradbury et al., 1991). Specific cAMP-BPs were identified in all the tissues examined and were of three

**Figure 3** Correlation between RI expression in tumour centre and tumour periphery; $r = 0.725$, $P < 0.001$. Line represents $x = y$.

**Figure 4** Correlation between RI expression in adjacent and distant mucosa; $r = 0.58$, $P < 0.01$. Line represents $x = y$.

**Figure 5** Correlation between RI expression in tumour periphery and adjacent mucosa; $r = 0.51$, $P < 0.05$. Line represents $x = y$.

**Figure 6** Scatter plot of RI expression in tumour centre, tumour periphery and distant mucosa expressed as a ratio of percentage RI expression in adjacent mucosa from the same patient. Horizontal lines represent median values. Only three specimens of tumour centre and six specimens of tumour periphery contained less RI than adjacent mucosa taken from the same patient. In contrast, there was no consistent difference between adjacent and distant mucosa.

main types with molecular weights of 48, 50 and 52 kDa. Other species of low molecular weight were also detected but constituted less than 15% of the total cAMP-BPs present. These smaller molecules have been detected by other workers and are generally considered to be degradation products (Rannels & Corbin, 1979). Immunoprecipitation with specific anti-RI and anti-RII antibodies showed that the 48 kDa moiety was a form of RI, while both the 50 kDa and 52 kDa moieties were forms of RII. The relationship of RII-50 to RII-52 is unknown. It is possible that they represent the dephosphorylated and autophosphorylated forms of RII respectively (Rangel-Aldao *et al.*, 1979). Alternatively, they may be separate gene products.

The second novel finding of the study is that there is significantly more RI-48 in both the centre and periphery of human colorectal cancers when compared with paired adjacent and distant benign mucosa. In contrast, RII-50 and particularly RII-52 were reduced in cancers. Malignant colorectal tissues therefore relatively overexpress RI-48 when compared with benign mucosa. To our knowledge, the only other published work on R-subunit expression in colorectal tissues was performed by Tagliaferri *et al.* (1988) on a human cell line LS-174T. This was found to express predominantly a 47 kDa RI protein but also to express lesser amounts of 52 and 54 kDa RII proteins. Interestingly, treatment with the cAMP analogue 8-Cl-cAMP resulted in an inhibition of growth and the promotion of morphological differentiation. These effects were associated with a reduction in RI but an increase in RII expression within the cell line. The relationship between these RII isoforms and the RII isoforms detected in the present study is unknown. However, the same antibodies were used for detection and so it is possible that they are, in fact, the same proteins and that differences in technique account for the small differences observed in molecular weight. Although cell lines have been relatively well investigated with regard to cAMP-binding protein patterns, to our knowledge this is the first study to have examined the pattern of cAMP-binding proteins within a resected human cancer. Other workers have looked at the expression of kinase activity within other types of human tumours, and the results from these studies would appear to support the observations made in this paper. Fossberg *et al.* (1978) found the PKAI/II ratio within a human renal cell carcinoma to be approximately twice that found in normal renal cortex. Yasui *et al.* (1985) found that while normal human gastric mucosa had a PK-AI/II ratio of 0.2, in xenotransplantable human gastric carcinomas it was approximately 0.6, leading them to conclude that type I PK-A was a biochemical marker for malignant transformation. The present work also shows that RI is particularly highly expressed in tumour of poor histological grade. This is in contrast to the studies of Fossberg *et al.* (1978) and Yasui *et al.* (1985), who were unable to demonstrate any association between type I PK-A kinase activity and histological grade. Taken together, these results and the present study provide evidence for excess PK-AI and RI expression in three separate human malignancies (renal, gastric, colorectal). It is possible, therefore, that such overexpression is a general phenomenon associated with carcinogenesis.

The present study also demonstrates a strong correlation between RI expression in tumour and mucosa from the same patient, suggesting that the relative overexpression of RI is superimposed upon a background level of RI expression which is highly variable between different individuals. It is interesting to speculate whether these differences reflect differences in cell proliferation as a hyperproliferative mucosa is generally proposed as the first detectable step in the process of colorectal carcinogenesis and is believed to affect the entire colon (Terpstra *et al.*, 1987).

In conclusion, this study is the first to have characterised cAMP-binding protein expression in human colorectal tissues and has demonstrated that resected human colorectal cancers, like human colon cancer cell lines, relatively overexpress RI cAMP-binding protein. Furthermore, a high level of RI expression is associated with poor histological grade. The observation that cAMP analogues can inhibit the growth and promote the differentiation of human colon cancer cell lines *in vitro* together with a reduction in RI expression suggests that such analogues may be able to exert biological control of colorectal cancers *in vivo*.

A.W. Bradbury was supported by the Syme Surgical Scholarship, Medical Faculty, Edinburgh, Scotland.

### References

BALL, R.L., TANNER, K.D. & CARPENTER, G. (1990). Epidermal growth factor potentiates cyclic AMP accumulation A-431 cells. *J. Biol. Chem.*, **265**, 12836–12845.

BRADBURY, A.W., MILLER, W.R. & CARTER, D.C. (1991). Cyclic adenosine 3', 5'-monophosphate binding proteins in human colorectal cancer and mucosa. *Br. J. Cancer*, **63**, 201–203.

BRADFORD, M.M. (1976). A rapid and sensitive method for the quantification of microgram quantities of protein utilizing the principle of protein dye binding. *Anal. Biochem.*, **72**, 248–253.

CHO-CHUNG, Y.S. (1989). Site selective 8-chloro-cyclic adenosine 3':5'-monophosphate as a biological modulator of cancer: restoration of normal control mechanisms. *J. Natl Cancer Inst.*, **81**, 982–987.

CHO-CHUNG, Y.S., TORTORA, G. & CLAIR, T. (1988). Nuclear location signal and nuclear translocation of type II protein kinase regulatory subunit in site-selective cAMP analog induced growth control and differentiation. *J. Cell Biol.*, **107**, 492a.

DECAMILLI, P., MORETTE, M., DONINI, S.D., WALTER, U. & LOHMANN, S.M. (1986). Heterogeneous distribution of the cAMP receptor protein RII in the nervous system: evidence for its intra-cellular accumulation on microtubules, microtubule organizing centres, and in the area of the Golgi complex. *J. Cell Biol.*, **103**, 189–203.

DUKES, C.E. (1932). The classification of cancer of the rectum. *J. Pathol.*, **616**, 315–321.

FOSSBERG, T.M., DOSKELAND, S.O. & UELAND, P.M. (1978). Protein kinase in human renal cell carcinoma and renal cortex. *Arch. Biochem. Biophys.*, **189**, 372–381.

GENTLEMAN, S., HEMMINGS, B.A., RUSSELL, P. & CHADER, G.J. (1989*a*). Developmental expression of the RI subunit of cyclic AMP-dependent protein kinase in the retina. *Exp. Eye Res.*, **48**, 717–731.

GENTLEMAN, S., HEMMINGS, B.A., RUSSELL, P. & CHADER, G.J. (1989*b*). Abnormal expression of the RI subunit of cAMP-dependent proteinkinase in Y-79 retinoblastoma cells. *Exp. Eye Res.*, **48**, 497–507.

GHARRET, G., MALKINSON, A.M . & SHEPPARD, J.R. (1976). Cyclic AMP-dependent protein kinases from normal and SV40-transformed 3T3 cells. *Nature*, **264**, 673–675.

HADDOX, M.K., ROESK, W.R. & RUSSELL, D.H. (1979). Independent expression of cardiac type I and II cyclic AMP-dependent protein kinase during murine embryogenesis and post-natal development. *Biochim. Biophys. Acta*, **585**, 527–534.

JAHNSEN, T., HEDIN, L., KIDD, V.J., BEATTIE, W.G., LOHMANN, S.M., WALTER, U., DURICA, J., SCHULTZ, T.Z., SCHILTZ, E., BROWNER, M., LAWRENCE, C.B., GOLDMAN, D., RATOOSH, S.L. & RICHARDS, J.S. (1986*a*). Molecular cloning, cDNA structure, and regulation of the regulatory subunit of type II cAMP-dependent protein kinase from rat ovarian granulosa cells. *J. Biol. Chem.*, **261**, 12352–12361.

JAHNSEN, T., HEDIN, L., LOHMANN, S.N., WALTER, U. & RICHARDS, J.S. (1986*b*). The neural type II regulatory subunit of cAMP-dependent protein kinase is present and regulated by hormones in the rat ovary. *J. Biol. Chem.*, **261**, 6637–6639.

LEE, D.C., CARMICHAEL, D.F., KREBS, E.G. & MCKNIGHT, G.S. (1983). Isolation of cDNA clone for the type I regulatory subunit of bovine cAMP-dependent protein kinase. *Proc. Natl Acad. Sci. USA*, **80**, 3608–3612.

LINASK, K.K. & GREENE, R.M. (1989). Subcellular compartmentalization of cAMP-dependent protein kinase regulatory subunits during palate ontogeny. *Life Sci.*, **45**, 1863–1868.

LORIMER, I.A., MASON, M.E. & SANWAL, B.D. (1987). Levels of type I cAMP-dependent protein kinase regulatory subunit are regulated by changes in turnover rate during skeletal myogenesis. *J. Biol. Chem.*, **262**, 17200–17205.

OYEN, O., MYKLEBUST, F. & SCOTT, J.D. (1989). Human testis cDNA for the regulatory subunit RII-alpha of cAMP-dependent protein kinase encodes an alternate aminoterminal region. *FEBS Lett.*, **246**, 57–64.

POMERANTZ, A.H., RUDOLPH, S.A., HALEY, B.E. & GREENGARD, P. (1975). Photoaffinity labelling of a protein kinase from bovine brain with 8-azidoadenosine 3′:5′-monophosphate. *Biochemistry*, **14**, 3858–3862.

RANGEL-ALDAO, R., KUPIEC, J.W. & ROSEN, O.M. (1979). Resolution of the phosphorylated and dephosphorylated cAMP binding protein of bovine cardiac muscle by affinity labelling and two-dimensional electrophoresis. *J. Biol. Chem.*, **254**, 2499–2508.

RANNELS, S.R. & CORBIN, J.D. (1979). Characterization of small cAMP-binding fragments of cAMP-dependent protein kinases. *J. Biol. Chem.*, **254**, 8605–8610.

SANDBERG, M., LEVY, F.O., OYEN, O., HANSSON, V. & JAHNSEN, T. (1988). Molecular cloning, cDNA structure and deduced amino acid sequence for the hormone-induced regulatory subunit (RII-beta) of cAMP-dependent protein kinase from rat ovarian granulosa cells. *Biochem. Biophys. Res. Comm.*, **154**, 705–711.

SANDBERG, M., SKALHEGG, B. & JAHNSEN, T. (1990). The two mRNA forms for the type I-alpha regulatory subunit of cAMP-dependent protein kinase from human testis are due to the use of different polyadenylation site signals. *Biochem. Biophys. Res. Comm.*, **167**, 323–330.

SCOTT, J.D., GLACCUM, M.B., ZOLLER, M.J., UHLER, M.D., HELFMAN, D.M., MCKNIGHT, G.S. & KREBS, E.G. (1987). The molecular cloning of a type II regulatory subunit of the cAMP-dependent protein kinase from rat skeletal muscle and mouse brain. *Proc. Natl Acad. Sci. USA*, **84**, 5192–5196.

SIKORSKA, M., WHITFIELD, J.F. & WALKER, P.R. (1988). The regulatory and catalytic subunits of cAMP-dependent protein kinases are associated with transcriptionally active chromatin during changes in gene expression. *J. Biol. Chem.*, **263**, 3005–3011.

SUPPATONE, S., DANOFF, S.K., THEIBERT, A., JOSEPH, S.K. & SNYDER, S.H. (1988). Cyclic AMP-dependent phosphorylation of a brain inositol triphosphate receptor decreases its release of calcium. *Proc. Natl Acad. Sci. USA*, **85**, 8747–8750.

TAGLIAFERRI, P., KATSAROS, D., CLAIR, T., ALLY, S., TORTORA, G., NECKERS, L., BOANERGES, R., PARANDOOSH, Z., CHANG, Y., REVANKAR, G., CRABTREE, G.W., ROBINS, R.K. & CHO-CHUNG, Y.S. (1988). Synergistic inhibition of growth of breast and colon human cancer cell lines by site-selective cyclic AMP analogs. Cancer Res., **48**, 1642–1650.

TERPSTRA, O.T., VAN BLANKENSTEIN, M., DEES, J. & EILERS, G.A.M. (1987). Abnormal pattern of cell proliferation in the entire colonic mucosa of patients with colonic adenoma or cancer. *Gastroenterology*, **92**, 742–748.

TOWBIN, H., STAEHELIN, T. & GORDON, J. (1979). Electrophoretic transfer of proteins from polyacrylamide gels to nitrocellulose sheets: procedure and some applications. *Proc. Natl Acad. Sci. USA*, **76**, 4350–4354.

WALSH, D.A., PERKINS, J.P. & KREBS, E.G. (1968). An adenosine 3′:5′-monophosphate dependent protein kinase from rabbit skeletal muscle. *J. Biol. Chem.*, **243**, 3763–3765.

WU, J.C. & WANG, J.H. (1989). Sequence-specific DNA binding to the regulatory subunit of cAMP-dependent protein kinase. *J. Biol. Chem.*, **264**, 9989–9993.

YASUI, W., SUMIYOSHIO, H., OCHIAI, A., YAMAHARA, M. & TAHARA, E. (1985). Type I and II cyclic adenosin 3′:5′-monophosphate-dependent protein kinase in human gastric mucosa and carcinomas. *Cancer Res.*, **45**, 1565–1568.